# Using Autumnal Trap Crops to Manage Tarnished Plant Bugs (*Lygus lineolaris*)

**DOI:** 10.3390/insects13050441

**Published:** 2022-05-07

**Authors:** François Dumont, Caroline Provost

**Affiliations:** Centre de Recherche Agroalimentaire de Mirabel, 9850 Rue de Belle-Rivière, Mirabel, QC J7N 2X8, Canada; cprovost@cram-mirabel.com

**Keywords:** trap cropping, integrated pest management, overwintering, landscape diversification

## Abstract

**Simple Summary:**

The tarnished plant bug *Lygus lineolaris* (Hemiptera: Miridae) is an important pest in several cultures. Trap crops are useful during the production season but could also contribute to the preventive regulation of *L. lineolaris* in autumn. The study aimed to test the attractiveness of the mullein plant as an autumnal trap crop and three repressive methods applied to this host. During autumn, mullein plants are a very attractive trap crop resulting in an aggregation of the *L. lineolaris*. Application of insecticide of the autumnal trap crop reduced winter survivorship of the pest. The combination of autumnal trap crop and insecticide is a promising strategy that could contribute to reducing *L. lineolaris* population during the following growing season.

**Abstract:**

For insects, surviving winter depends on their capacity to store enough energy and find proper hibernation sites. A common strategy is to minimize movement and hibernate near autumn food sources. We investigated the efficiency of autumnal hosts to act as trap crops where insects could be exposed to targeted repressive treatments. This approach could reduce the local populations of insect pests in the next production season, reducing the need for insecticides. First, we tested the mullein plant’s attractiveness as an autumn trap crop for *Lygus lineolaris* (Hemiptera: Miridae) in strawberry fields by comparing peak population density among mullein (*Verbascum thapsus*), strawberry plants (*Fragaria* × *ananassa*), buckwheat (*Fagopyrum esculentum*), and mustard (*Sinapis alba*). Second, we tested four treatments applied to the autumn trap crops to reduce *L. lineolaris* winter survivorship: (1) hot water, (2) a pathogen (*Beauveria bassiana*), (3) insecticide (cypermethrin), and (4) a control. The density of the *L. lineolaris* population on mullein in autumn and on buckwheat in summer was higher than on strawberry and mustard. Of the overwintering *L. lineolaris*, 0% survived the winter when treated with the insecticide cypermethrin, while 38.3% survived in the control treatment (without repressive treatment). The *B. bassiana* and hot water treatments did not differ from the control. The mullein autumn trap crops combined with insecticide treatments could contribute to reducing the overwintering population, hence potentially reducing population during the following growing season.

## 1. Introduction

Insects are pests when they inhibit the production of crops. A strategy is to use trap crops to attract, divert, intercept, and retain pest insects away from the main crops [1]. Aggregation on the trap crops offers the opportunity to target these high-density locations with repressive treatments [2,3]. Usually, trap crops are used to attract pests by providing food resources and egg-laying sites to protect the main crops during the growing season [4]. However, trap crops could theoretically be designed to attract pest insects at any time of the year. We suggest that trap crops could be used to provide a site for pests, where aggregation would enhance the efficiency of repressive methods and limit further outbreaks. Winter survival is a determining factor in the size of the population in spring [5]. There is little empirical evidence to demonstrate the effectiveness of autumn trap crops (hibernation sites) combined with repressive methods (e.g., insecticides or biological control agents) as a preventive method to control pest insects.

In temperate climatic zones, overwintering insects use diapause to cope with the rigors of winter through a negative phototaxis response [6,7,8]. During a pre-diapause period, insects accumulate energy [9,10,11] and gather at suitable hibernation sites [8,12]. Moreover, their level of activity decreases as the temperature falls [7,12]. Due to their aggregation at hibernation sites and limited activity, insects in a pre-diapause period would be intensively exposed to repressive approaches such as chemical or biological control. In addition to direct mortality, repressive interventions could indirectly decrease the survival of the overwintering insects by forcing them to invest their energy reserves in defense mechanisms against the threats (repressive methods), thereby creating a trade-off in energy investment. For instance, the physiological resistance to insecticides requires an energy investment [13]. Resources allocated to defense against repressive methods reduce the energy available for overwintering and could jeopardize winter survival [9]. Directly and indirectly, repressive methods targeted in such trap crops could be highly useful to prevent outbreaks in the following spring.

Tarnished plant bugs (TPB) *Lygus* spp. are major pests in crops worldwide [3,14,15]. These pests can be effectively managed using summer trap crops [2,3]. For instance, alfalfa (*Medicago sativa* L.; Fabaceae) blooms early in the season and attracts the western tarnished plant bugs *Lygus hesperus* (Knight) (Hemiptera: Miridae) [2,16,17]. Similarly, white mustard (*Sinapis alba* L.; Brassicaceae) and buckwheat (*Fagopyrum esculentum* Moench; Polygonaceae) is attractive to the tarnished plant bugs (TPB) *Lygus lineolaris* (Palisot de Beauvois) (Hemiptera: Miridae) during summer [3]. In the autumn, the tarnished plant bugs exploit different hosts than during the summer, in response to blooming seasonality [18]. Mullein plants (*Verbascum thapsus*, Scrophulariaceae) are among the most interesting hibernation site for the tarnished plant bug. This host’s pubescent leaves offer a quality resource [19,20] and a refuge for tarnished plant bugs in hibernation. Hence, mullein plants could be useful as autumn trap crops.

Both chemical [21,22,23] and biological insecticides [24,25,26] could repress tarnished plant bug populations. Various strains of the entomopathogenic fungus *Beauveria bassiana* are lethal to tarnished plant bugs [25,26,27,28]. While lower temperatures, higher humidity, and reduced sun exposure during autumn could reduce germination and growth of *B. bassiana*, it could also decrease TPB immune response [29]. Moreover, the resource allocated to the immune defense would not be available to invest in stored energy that increases winter survivorship [29]. Similarly, the autumn application of chemical insecticides could compromise the insect’s physiological and behavioral responses to cope with or avoid these chemicals. This would decrease the winter survivorship of the pest.

In this study, we tested the mullein plants’ efficiency as autumnal trap crops to manage the tarnished plant bugs. This preventive strategy relies on two elements: (1) the density of targeted insects in the trap crops and (2) the effectiveness of repressive methods to reduce the winter survival of pests. Therefore, we compared the density of the tarnished plant bugs observed during the peak on each host among strawberry plants, buckwheat, mustard, and mullein. Then, we compared the efficacy of three repressive methods (boiling water, entomopathogenic fungus, and insecticide) to reduce the winter survivorship of tarnished plant bugs.

## 2. Materials and Methods

### 2.1. Study Site

We conducted our field experiments on the experimental farm at the Centre de recherche agroalimentaire de Mirabel (Mirabel, QC, Canada) (45.648934° N, −74.090042° E). We monitored the tarnished plant bug population during summer and autumn 2016 and 2017. The winter survivorship experiment was conducted during 2016–2017 and 2017–2018.

### 2.2. Experimental Design and Data Collection

#### 2.2.1. Summer Trap Crop Plots

**Figure 1:**
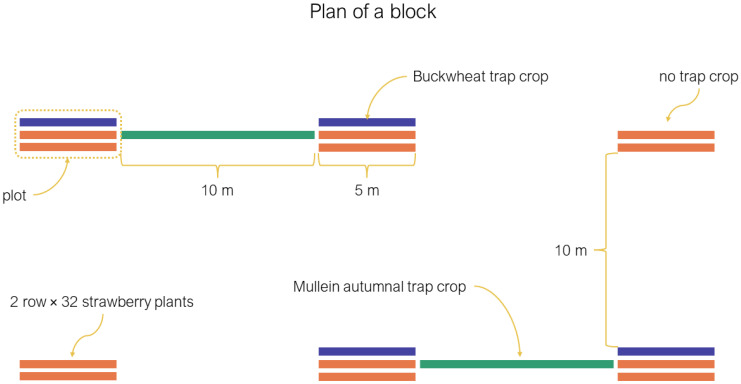
Experimental design plan of a typical block.

During the summers of 2016 and 2017, we planted four blocks of six plots. Each plot contained two rows (5 m × 1 m) of 32 strawberry plants each (variety “Albion”) and four of those plots also had one adjacent row of buckwheat (*Fagopyrum esculentum* Moench; Polygonaceae) or white mustard (*Sinapsis alba* L.; Brassicaceae) (Figure 1). The position of the summer treatments was randomized within blocks. The density of buckwheat and mustard was 65 plants/m^2^ in both years (about 325 plants per trap crop). We planted transplants of strawberries and the summer trap crops at the end of May. Plots were separated by 10 m, and blocks by 20 m. The summer experimental design was part of another research project, partly published in Dumont and Provost [3].

We monitored the summer populations of tarnished plant bugs in these plots weekly from July to September by beating two strawberry plants per row and two (2016) or four (2017) seedlings of buckwheat or mustard per row. The beating consisted of three gentle “beats” per plant using the hand over a white cloth. We counted the TPB (both adults and nymphs).

#### 2.2.2. Autumn Trap Crop Plots

We planted two trap crop plots of mullein plants per block between the summer plots (see Figure 1). Each mullein trap crop was composed of 20 vegetative mullein plants (first-year plant) arranged over 10 m, the distance between the summer plots (Figure 1). The plants were grown in a greenhouse and planted outside in early July on plastic mounds. The mullein plants’ seeds were harvested from wild plants.

We monitored the tarnished plant bug population on mullein plants weekly from the end of August through to the end of October. We thoroughly inspected five mullein plants per plot (visual inspection). All leaves of the mullein plants were inspected on both sides. It took about 2 min per sample. A single person conducted all the observations. The visual inspection was used instead of the beating because the latter method can hardly be applied to plants shaped like mullein plants. Both methods properly estimated the density of TPB on each plant species involved in this experiment.

#### 2.2.3. Winter Survivorship

We evaluated the TPB winter survivorship on the mullein plants. In late October (25 October 2016 and 2017), we captured tarnished plant bugs on the mullein plants using an entomological vacuum and cleaned those plants of all other insects by removing these by hand with a brush. We then installed 60 (2016) and 40 (2017) net cages (30 cm × 30 cm × 30 cm) over the mullein plants. There was only one plant per cage. We introduced four adult tarnished plant bugs in each of those cages. The cages were evenly distributed in the eight plots from the trap crops experiment. We randomly applied one of four treatments to each of the cages: (1) a control treatment without repressive treatment; (2) 2 L of boiling water poured on the plants after the first snowfall on 12 December both years; (3) bioinsecticide (*B. bassiana*) (0.5 L per plant with a dilution of 1 mL/5 L); and (4) Ripcord 400 insecticide (i.e., cypermethrin) (0.7 L per plant with a 1 mL/7 L dilution). Each treatment was replicated 25 times. Both insecticide treatments (3 and 4) were applied in early November when the daily maximum temperature was about 10 °C. In mid-December, when the bugs were in diapause, we removed the cages’ lids to allow the snow to enter the cages. We removed the lids when the tarnished plant bugs were inactive and under the snow. The snow plays a role in insulation, allowing the survival of the tarnished plant bugs. The lids were put back on the cage before all the snow melted, on 10 April both years. We opened the cages on 27 April both years to inspect the plants and count the tarnished plant bugs.

### 2.3. Statistical Analysis

#### 2.3.1. Population Density on Mullein Plant

We tested the linear and quadratic pattern of weekly variations of tarnished plant bugs (both adults and nymphs) on mullein plants in autumn with a generalized linear mixed model (GLMM) for Poisson-distributed data. The random effects included the block nested in the year. These random effects were tested by comparing models with and without each variable, using an analysis of variance (ANOVA).

#### 2.3.2. The Peak of Population Density on Summer and Autumnal Hosts

The peak of tarnished plant bug density (all stage pooled) was identified for each host for each year. The peak period was defined as the week with a higher density of tarnished plant bugs for each plant and the weeks before and after that week. Hence, for each host, the dataset includes three weeks of observation for each year.

A generalized linear mixed model (GLMER) for Poisson-distributed data was used to compare the density of tarnished plants during each host’s peak period. The host was the fixed effect in the model, whereas the plot, the block, and the year were included as random effects.

#### 2.3.3. Winter Survivorship

The winter survival rate of tarnished plant bugs was tested using a GLMM. The repressive treatment was included as a fixed effect. In the insecticide treatment, no tarnished plant bug survived the winter. Thus, this treatment could not be included in the statistical analyses due to the absence of variance in the observations. The block nested in the year was included in the model as a random variable to consider the proximity of some treatments and the effect of the year. Then, the 95% confidence interval was calculated for the treatment using the Wald method. The control, *B. bassiana*, and water treatments were considered different from the insecticide treatment if the intervals did not include 0 (the actual value of the insecticide treatment).

## 3. Results

### 3.1. Population Density on Mullein Plant

**Figure 2:**
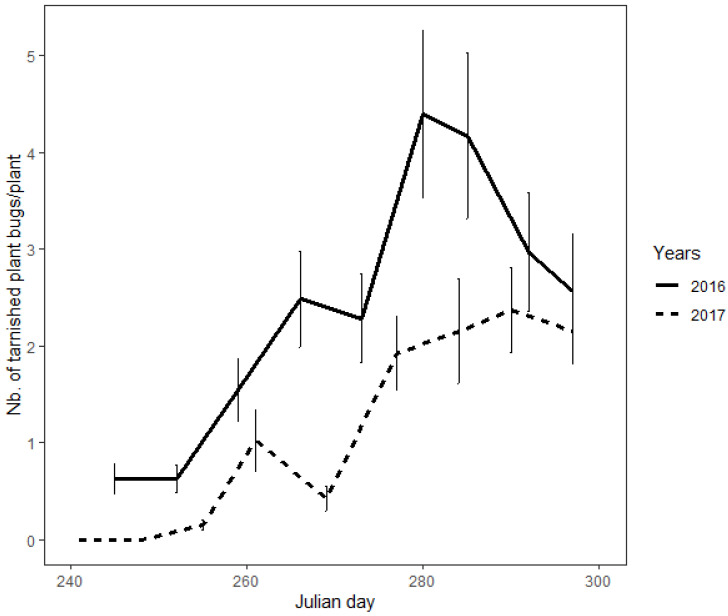
Seasonal variation of the tarnished plant bug population on mullein plants during 2016 and 2017.

In autumn, the density of tarnished plant bugs on mullein plants increased according to both linear (β = 23.85 ± 1.41; z = 16.91; *p* < 0.0001) and quadratic (β = −10.66 ± 1.07; z = −9.94; *p* < 0.0001) patterns (Figure 2). The highest densities were 4.43 (±0.87 s.e.) and 2.43 (±0.44) individuals per plant in 2016 and 2017, respectively. The year (χ^2^ = 100.28; df = 1; *p* < 0.0001) and the block (χ^2^ = 393.9; df = 1; *p* < 0.0001) were both statistically significant, indicating that there were more tarnished plant bugs in trap crops in 2016 than in 2017, and that tarnished plant bugs were not equally distributed between the fields. Tarnished plant bugs were observed on 51.5% of the sampled mullein plants. On plants where tarnished plant bugs were present, groups of two or more individuals were observed in 67.8% of the cases. A maximum of 22 individuals were observed on the same mullein plant.

### 3.2. The Peak of Population Density on Summer and Autumnal Hosts

**Figure 3:**
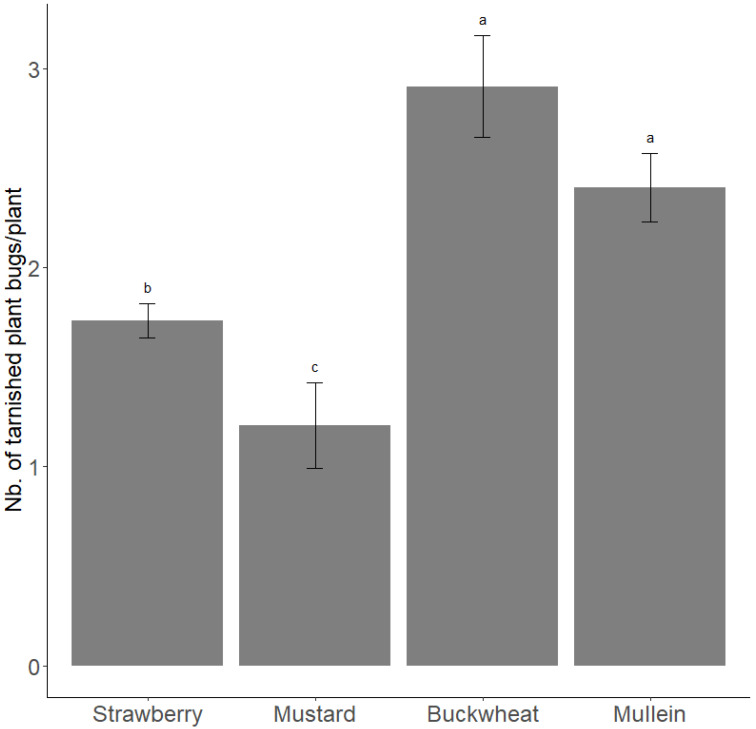
Density of tarnished plant bugs (all stage pooled) during period of peaks on hosts (strawberry plant, mustard, buckwheat, and mullein) during 2016 and 2017. Estimated number of adult tarnished plant bugs during peak of population from beatings on strawberry, mustard, and buckwheat during summer (in light grey) and on mullein during autumn (in dark grey) in 2016 and 2017. Error bars are 95% confidence intervals. Letters correspond to significant differences among treatments (α = 0.05).

In summer 2016, the peak density of tarnished plant bugs (all stage pooled) on mustard, buckwheat, strawberry plants, and mullein was on 20 July, 4 August, 8 September, and 6 October, respectively. A similar trend was observed in 2017 with a peak density on 15 August, 4 September, and 17 October, respectively, for the buckwheat, strawberry plants, and mullein. On average over the two years, the density of tarnished plant bugs during each host’s peak period was higher on buckwheat and mullein and lower on mustard than on strawberry plants (LRT_3_ = 128.06; *p* < 0.0001) (Figure 3).

### 3.3. Winter Survivorship

**Figure 4:**
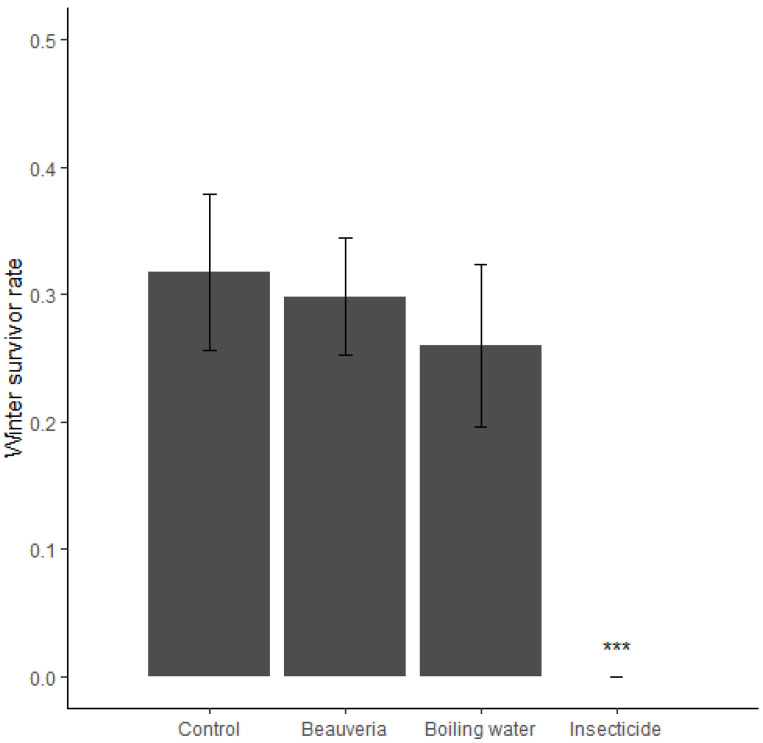
Winter survivorship rate of the tarnished plant bugs (calculated as proportion of surviving bugs on the number of introduced individuals) as a function of the repressive treatment. Error bars correspond to 95% confidence intervals. The “***” indicates that the treatment is statistically different than the other treatments.

The mean winter survival rate was 0.32 (95% CI: 0.23; 0.42) in the control treatments (Figure 4). *B. bassiana* (mean = 0.30; 95% CI: 0.21; 0.40) and boiling water (mean = 0.26; 95% CI: 0.17; 0.36) treatments had no significant effect on winter survivorship of tarnished plant bugs (LRT_2_ = 0.62, *p* = 0.73) (Figure 4). No living bugs were observed in the spring in any cage of the insecticide treatment. This treatment could not be included in the statistical analyses. The confidence interval (95% CI) from the control, the *B. bassiana*, and boiling water treatments all excluded 0 (the value of the insecticide treatment).

## 4. Discussion

The summer trap crops are designed to divert insect pests from crops [1]. In contrast, autumn trap crops are preventative methods aiming to take advantage of insects’ vulnerability at low temperatures to reduce their winter survivorship. First, this approach’s relevance depends on the trap crop’s ability to attract high densities of the pest. Thus, a minimum of repressive efforts can eliminate a maximum of pests. The density of tarnished plant bug population per mullein plant increased late in the season, from the beginning of September to about mid-October. During the peak period (beginning to mid-October), the densities of tarnished plant bugs per plant observed on mullein were similar to the densities observed in summer on buckwheat but higher than on mustard and strawberries. Second, for our approach to be relevant, repressive treatments applied to mullein plants must effectively eliminate insect pests. In our experiment, *Beauveria* and boiling water treatments did not affect tarnished plant bugs’ survivorship during the winter. However, the insecticide treatment (cypermethrin) was 100% effective: no bugs survived winter after being exposed to this treatment. This high efficacy of insecticides suggests that tarnished plant bugs are vulnerable at this time of the year because they are aggregated, accessible, and have low mobility. Thus, our study confirms the potential of the autumn trap crops combined with insecticides as a preventative approach in the control of tarnished plant bugs. The medium and long-term consequences of this method, however, remain to be determined.

The scarcity of quality host plants during autumn (resource concentration theory), density-dependent anti-predator behaviors (e.g., encounter, dilution, and selfish herd effects), or other benefits of the group (e.g., thermoregulation) can explain the high density of tarnished plant bugs on mullein plants. Tarnished plant bugs exploit different hosts from spring to autumn [18,30]. During autumn in Quebec, the vegetation is affected by the short length of days and cold temperatures (an average of approximately 10 °C in October). Therefore, the number of host plants available for tarnished plant bugs decreases considerably. Strawberry plants generally start a dormant period in October. In contrast, the biannual mullein plant leaves remain green all year long and are a suitable host for Mirid bugs [19,20]. Thus, the mullein trap crops contrast with their immediate environment by offering a high concentration of resources in an environment where these are rare. In addition, cold temperatures in autumn in Quebec reduce the mobility of tarnished plant bugs. This reduced mobility could increase the vulnerability of tarnished plant bugs to predation [31]. In a vulnerable situation, insects can use density-dependent anti-predation strategies (i.e., dilution, encounter, and selfish herd effects) [32,33,34,35]. For example, the risk of predation decreases proportionally with the size of the group [33,34]. In our experiment, we observed groups of up to a maximum of 22 individuals per plant. These dense groups could offer an advantage over predators compared to bugs that find themselves alone on the plants.

The insecticide (cypermethrin) applied to mullein plants in the autumn trap crop was very efficient, killing 100% of the bug population. In Dumont and Provost [3], the summer application of cypermethrin on strawberry, buckwheat, and mustard plants killed less than 50% of the tarnished plant bug population. The tarnished plant bugs develop behavioral and physiological resistance to various insecticides [22,36,37]. The insects may be more exposed to the insecticide in autumn than in summer, increasing its efficiency. Behavioral resistance to insecticides will occur if insects avoid the treated area by moving to an untreated area [38]. In our experiment, we caged the tarnished plant bugs on the autumn trap crops preventing escape to a non-toxic place. However, we would expect similar results under uncaged conditions since the cold outside temperatures (close to 0 °C) limit the tarnished plant bugs’ mobility.

Physiological resistance to insecticides is energetically costly, with a consequence on fitness [13,39]. Resisting requires energy when it relies on metabolic detoxification or excretion/increased impermeability of membranes [39]. In autumn, energy storage is based on stored fat reserves accumulated in the season and is limited. When the energy is used to engage insecticide resistance mechanisms, it is not available to ensure energetic demands of overwinter survival. As a result, insects subjected to insecticide treatment in our experiment had to trade off energy between resistance to insecticide and winter survival. Our results suggest that this trade-off is not viable for the tarnished plant bugs during winter when food resources are unavailable. In the tarnished plant bug, insecticide resistance increased over the warm season (spring to autumn) but decreased during winter, suggesting a trade-off between winter survival and insecticide resistance [36]. In the summer, tarnished plant bugs would have the mobility to avoid treated areas until the insecticide is degraded, and the energy to sustain physiological resistance demands. Thus, targeting the tarnished plant bugs with insecticides in the autumn might be a more effective control approach.

In our study, the entomopathogenic fungus *B. bassiana* applied during autumn did not increase winter mortality. *B. bassiana* treatments may induce lower mortality at low temperatures than at high temperatures, but with a similar inoculation rate [40] Hence, our *B. bassiana* treatments may have resulted in the tarnished plant bug inoculation without immediate mortality. The pathogenic fungus may affect individuals’ reproduction and long-term survival [41,42]. Ugine [42] observed that female tarnished plant bugs’ longevity and total egg production were negatively affected by *B. bassiana* treatments, resulting in a decrease in the intrinsic rate of natural increase. Such a reduction could have longer-term effects on the second generation [42]. In addition, the application of *B. bassiana* could have longer-term repercussions compared to insecticides if the fungus reproduces and spreads in the environment. Alternatively, the fungus might be dormant in the winter and induce similar lethal effects with the arrival of warmer temperatures. The effect on reproduction and longevity has not been measured in the time scale of our experiments. Therefore, more long-term assessments would be needed before rejecting this repressive approach.

## 5. Conclusions

The autumnal trap crop was very attractive to the tarnished plant bugs and provides an opportunity to target this pest with repressive methods. However, the combined use of trap crops and repressive methods can have adverse effects on other organisms, including predators. In our study, we also observed the presence of the damsel bug *Nabis americoferus* (Carayon) (Hemiptera: Nabidae) on several of the mullein plants. Moreover, this tarnished plant bug predator could be responsible for the decline in the population of tarnished plant bugs observed on mullein plants at the end of October. However, damsel bugs would be as vulnerable as tarnished plant bugs to repressive treatments if they use mullein for hibernation. The impact on their population should be considered before using specific repressive methods such as insecticides. Alternatively, the presence of damsel bugs suggests that this predator could be used for the biological control of tarnished plant bugs.

## Data Availability

The data presented in this study are openly available in Zenodo at https://doi.org/10.5281/zenodo.6524410.
